# Activity of the Gamma Secretase Inhibitor AL101 in Desmoid Tumors: A Case Report of 2 Adult Cases

**DOI:** 10.3390/curroncol28050312

**Published:** 2021-09-21

**Authors:** David Chan, Jason Kaplan, Gary Gordon, Jayesh Desai

**Affiliations:** 1 Bill Walsh Translational Cancer Research Laboratory, Northern Clinical School, University of Sydney, Sydney, NSW 2065, Australia; david.chan@sydney.edu.au; 2 Clinical Development, Ayala Pharmaceuticals, Northbrook, IL 60062, USA; jason.k@ayalapharma.com (J.K.); gary.g@ayalapharma.com (G.G.); 3 Department of Medical Oncology, Peter MacCallum Cancer Centre Royal Melbourne Hospital, Melbourne, VIC 3000, Australia

**Keywords:** aggressive fibromatosis, AL102, BMS-986115, BMS-906024, Notch pathway, rare disease

## Abstract

Desmoid tumors (aggressive fibromatosis) are soft tissue mesenchymal tumors that can be locally invasive and life-threatening. Depending on the location, these tumors are often unresectable or tend to recur after surgery. To date, there are no approved systemic therapies for desmoid tumors. These tumors typically harbor mutations in the β-catenin oncogene *CTNNB1* or the tumor suppressor gene adenomatous polyposis coli, resulting in constitutive activation of the WNT pathway. The Notch pathway is part of the underlying cause for desmoid tumor development, possibly due to crosstalk with the WNT pathway, providing a rationale for Notch inhibition as a therapeutic strategy. The gamma secretase activation of the Notch receptor can be targeted with investigational gamma secretase inhibitors. In this case report, we follow the course of 2 patients with desmoid tumors treated with the highly potent, parenterally administered investigational gamma secretase inhibitor AL101, resulting in long-lasting responses. Case 1 reports on a patient with a mesenteric desmoid tumor who participated in a phase 1 trial and then transitioned into a compassionate use program; Case 2 reports on a patient with recurrent pelvic tumors receiving AL101 through a compassionate use program. After tumor progression on other systemic therapies, Cases 1 and 2 had confirmed partial responses (41% and 60% maximal tumor size decrease from baseline) recorded after 1.0 and 1.6 years of treatment with AL101, with a duration of response of 8.6+ and 2.6+ years, respectively. Also, in a phase 1 study of AL102, a potent orally administered gamma secretase inhibitor that shares structural features with AL101, a patient with a desmoid tumor was noted to have tumor shrinkage. Formal clinical testing of AL102 for the treatment of patients with desmoid tumors that are not amenable to surgery or are refractory to/recurrent from other prior therapies is currently underway.

## 1. Introduction

Desmoid tumors (DT), also called aggressive fibromatosis, are benign tumors without metastatic potential that have a variable and unpredictable course, ranging from indolent to locally invasive [1]. These soft tissue mesenchymal tumors may severely impact critical organs and are associated with a high rate of recurrence [2,3]. DT is rare [4], with an incidence of about 5 to 6 new cases per million people per year [2,5]. Although a period of active surveillance is often the frontline approach, surgery has been a primary therapy for resectable DT in cases of disease progression [2]. In patients who undergo complete resection, up to 20% to 30% experience disease recurrence, and patients may undergo repeated surgical resections, which are associated with a greater risk of morbidity [6,7]. Although cytotoxic chemotherapy, tyrosine kinase inhibitors (TKI), hormonal therapy, and nonsteroidal anti-inflammatory drugs are treatment options, there are no Food and Drug Administration (FDA)-approved therapies for patients with DT [8]. Because of the lack of FDA-approved treatment options and the complexity of treating the disease, patients with DT should be followed by an experienced multidisciplinary team of soft tissue sarcoma experts to ensure that these patients receive appropriate care [2].

Most cases of DT (~85%) arise as a result of sporadic mutations in the WNT pathway, mainly as activating somatic mutations in the β-catenin oncogene *CTNNB1* [9,10,11]. More rarely, inactivating germline mutations in the tumor suppressor gene adenomatous polyposis coli (*APC*), a negative regulator of the WNT/β-catenin pathway, lead to constitutive activation of the WNT pathway. This activation is associated with the hereditary cancer predisposition syndrome familial adenomatous polyposis (FAP) [12,13]. DT is typically found in patients with FAP [12]. 

Although it is believed that the Notch pathway is involved in the pathogenesis of DT, its exact role has yet to be determined [14]. Crosstalk between the Notch and WNT pathways may be involved in the development of disease [15], which provides a rationale for Notch inhibition in the treatment of patients with DT [14,15,16]. Gamma secretase inhibitors (GSIs) prevent the proteolytic cleavage of the Notch receptor, thereby preventing the release of the Notch intracellular domain fragment, which is the key step for activation of all downstream effects. This leads to decreased expression of a number of Notch target genes, including those in the HES family [16]. Results from recent clinical trials and case studies have indicated that GSIs have clinical activity in patients with DT [17,18,19,20]. AL101 and AL102 are structurally similar, potent allosteric small molecule inhibitors of gamma secretase (parenterally and orally administered, respectively) that abrogate activation of all 4 human Notch receptors [21,22]. In the phase 1 dose-escalation study of AL101 (CA216001; NCT01292655), 94 heavily pretreated patients with advanced solid tumors received intravenous doses of AL101; 3 patients with DT were enrolled, of whom 2 had confirmed partial responses (PRs) and 1 had stable disease [17]. In a phase 1 dose-escalation study (NCT00878189) of the GSI nirogacestat (PF-03084014), in which Notch-related target inhibition was observed, 9 patients with DT were enrolled (7 were evaluable for response): 5 had a PR and 2 had stable disease [19]. In a phase 2 study (NCT01981551) that investigated nirogacestat in 17 adults with unresectable and/or recurrent/refractory progressive DT, 5 patients (29%) achieved a PR [18].

AL101 binds to and inhibits the gamma secretase complex, preventing cleavage of the Notch receptor, thereby inhibiting activation of the Notch pathway [22]. AL101 as a monotherapy is currently being investigated in a phase 2 study in patients with adenoid cystic carcinoma with Notch-activating mutations (ACCURACY; NCT03691207) [23,24]. AL101 monotherapy is also being investigated in an ongoing phase 2 study (TENACITY; NCT04461600) in patients with Notch-activated metastatic triple-negative breast cancer who have received ≤3 lines of prior therapy for metastatic disease [25]. 

Here, we report the activity and safety of AL101 for 1 of the 3 patients with DT from the AL101 phase 1 study (NCT01292655), along with information on another patient with DT who received AL101 through a compassionate access program.

## 2. Case Presentation

### 2.1. Patient 1

**Figure 1:**
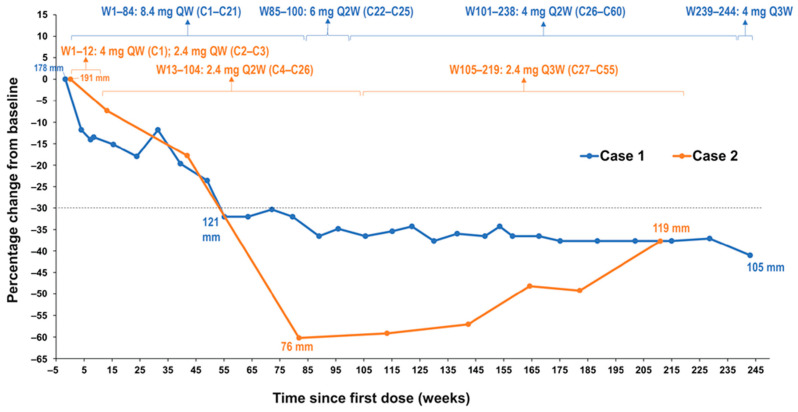
Change in desmoid tumor size from baseline in both patients by RECIST v1.1. Notes: Dotted line denotes change from baseline of 30%. Abbreviations: C, cycle; QW, once weekly; Q2W, once every 2 weeks; Q3W, once every3 weeks; Q4W, once every 4 weeks; W, week; RECIST, Response Evaluation Criteria in Solid Tumors.

**Figure 2:**
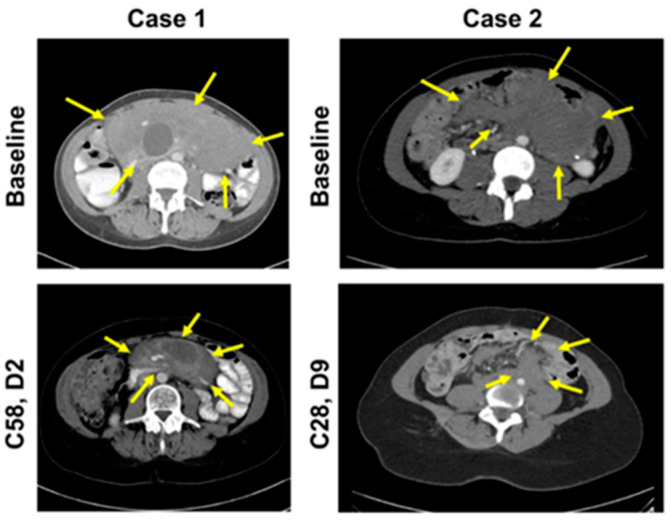
CT scans of target lesions at baseline and after achieving PR in both patients. Abbreviations: C, cycle; CT, computed tomography; D, day; PR, partial response.

In 2011, a 42-year-old female was diagnosed with a large mesenteric DT with a *CTNNB1* T41A somatic mutation (Table 1). The mesenteric tumor was intimately related to the superior mesenteric vessels, and due to its size and location, was not considered surgically resectable without major morbidity (sacrificing most of her small bowel and/or considering a small bowel transplantation). The patient was treated with imatinib with no response and then received 2 subsequent lines of therapy (tamoxifen and doxorubicin) without any evidence of an objective response to either agent. Approximately 14 months after her initial diagnosis, after failure of these therapies, the patient was enrolled into a phase 1 trial of AL101 at a dose of 8.4 mg once weekly (QW) on a 4-week cycle. AL101 was reasonably well tolerated; most adverse events were grade 1/2 and included diarrhea and fatigue. The diarrhea fluctuated but remained at a grade 1 or 2 level and was managed with antidiarrheals, when required. On cycle 21 day 22, the patient developed grade 3 diarrhea; therefore, because the diarrhea was intermittent before this but longstanding, the dose of AL101 was reduced to 6 mg QW for cycle 22. Grade 3 diarrhea was short-lived and resolved with intervention and dose modification. During cycle 26, the dose of AL101 was further reduced to 4 mg QW due to ongoing fatigue, lymphopenia, albuminemia, and intermittent episodes of diarrhea. The patient received AL101 during the clinical trial for approximately 4.6 years (60 cycles). At the close of the trial, the patient was transferred to a compassionate use program during which the patient received two additional doses. The patient achieved a PR per Response Evaluation Criteria in Solid Tumors (RECIST) version 1.1 at cycle 14, after approximately 1 year of treatment with AL101, and this response was maintained for more than 3.6 years while participating in the phase 1 trial (Figure 1 and Figure 2). The maximal response was a 41% decrease in the longest diameter tumor (a decrease from 178 mm to 105 mm). The patient elected to discontinue AL101 treatment after 4.6 years, with follow-up continuing. This decision was a personal choice made in consultation with the treating team. The decision was based, in part, on a plateau in disease response, burdensome ongoing intravenous therapy for which travel was required, and mild toxicities, but troublesome considering the good disease control. The prospect of restarting drug/rechallenging remained open to the patient. Hence, the decision to initially hold and then cease treatment was made. Off treatment, this patient has maintained a PR for an additional 4 years, with an overall duration of response of 8.6+ years. The patient remains on active surveillance. 

### 2.2. Patient 2

A 27-year-old female with a history of FAP (with a documented *APC* germline mutation) underwent a planned prophylactic total proctocolectomy and ileoanal pouch approximately 5.5 years ago. This surgery incidentally revealed the presence of multiple DTs (Table 1). A right iliac fossa mass was palpable on clinical follow-up 7 months after surgery and computed tomography imaging indicated the likely presence of a solitary 7-cm pelvic mass, which provided evidence for recurrent DT. On multidisciplinary review, biopsy of this tumor was thought to be high risk due to its location, and the diagnosis of recurrent DT was therefore made clinically and radiologically. Tamoxifen and 2 subsequent lines of chemotherapy (dacarbazine/doxorubicin, methotrexate/vinblastine) were administered without significant symptomatic or radiological response. Approximately 4.2 years ago (1 year and 4 months after diagnosis), AL101 therapy was therefore commenced on a compassionate access basis at a dose of 4 mg QW; however, the dose was reduced to 2.4 mg QW due to grade 2 elevated liver function tests. AL101 was further reduced to 2.4 mg every 2 weeks due to grade 3 diarrhea that resulted in dehydration, dizziness, and hospital admission. Grade 3 diarrhea had a sudden onset, occurring within 1-2 weeks of treatment initiation; it was short-lived and resolved with dose modification and without significant medical intervention. AL101 was subsequently reduced to 2.4 mg every 3 weeks because of fatigue and nausea, as well as the burden of coming into the clinic for regular injections. Imaging at cycle 21 day 13, approximately 1.5 years after AL101 initiation, revealed a PR (the longest diameter of tumor decreased from 191 mm to 76 mm, representing a 60% decrease) that has been maintained over the past 2.6 years (Figure 1 and Figure 2). The patient has continued treatment and the PR is ongoing. The patient reported improved quality of life, maintained throughout the duration of the treatment, with minimal side effects and short infusion time.

## 3. Discussion

In the 2 cases described here, one involving a patient in a phase 1 trial treated initially with AL101 8.4 mg QW with subsequent dose tapering to 4 mg Q2W, and the other involving a patient in an AL101 compassionate use program treated initially at 4 mg QW with subsequent dose tapering to 2.4 mg every 3 weeks, both patients presented with massive tumor burden, with symptomatic and life-threatening disease due to disease bulk and location. Both patients achieved sustained PRs with AL101 treatment, with a maximal decrease in tumor size from baseline of 41% after approximately 1 year (55 weeks) of treatment in Case 1 and a maximal decrease in tumor size from baseline of 60% after about 1.6 years (82 weeks) of treatment in Case 2. Both patients continue to maintain PRs. In Case 1, this PR has been maintained for a further 4 years (ongoing) since treatment cessation. Grade 3 diarrhea occurred in both patients; however, this adverse event was short-lived and resolved with dose modification. Appropriate prospective management of this side effect may be important for the optimal use of this drug going forward. Several other chronic toxicities were also observed; however, the patients responded well to dose and/or schedule modification, with ongoing treatment activity After participating in the phase 1 trial, the patient in Case 1 was given access to the compassionate use program, and at the patient’s request, AL101 treatment was discontinued. The patient in Case 2 remains on AL101 treatment.

**Figure 3:**
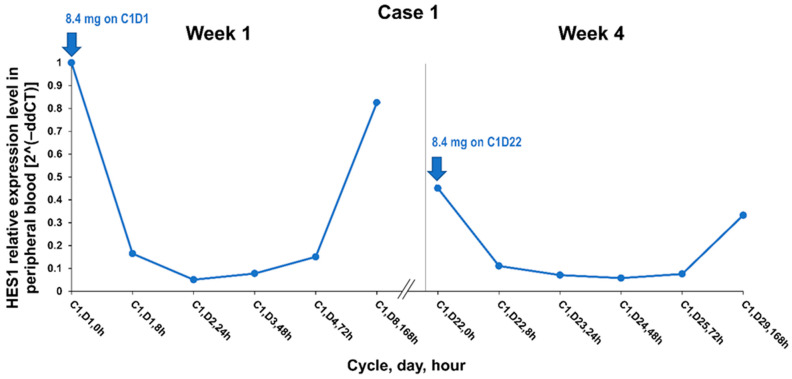
HES1 relative expression level at week 1 and week 4 of cycle 1 in Case 1. Abbreviations: C, cycle; D, day; h, hour.

Desmoid tumors frequently have mutations in *APC* or *CTNNB1*; however, mechanisms driving functional activation and tumor growth are not well understood [2]. As previously mentioned, there may be crosstalk between the WNT/β-catenin pathway and the Notch pathway, possibly mediated via transcription factor Hes1, which is encoded by *HES1*. The patient in Case 1 was diagnosed with a *CTNNB1* T41A somatic mutation, and the patient in Case 2 had an *APC* germline mutation. A 20-fold decrease in gene expression of *HES1*, a gene downstream in the Notch pathway, was noted 24 hours after the first dose of AL101 in the peripheral blood from the patient in Case 1 (Figure 3).

Oral TKIs have also shown promising results in clinical trials examining therapeutic options for patients with progressive and refractory DT [26,27]. A phase 3 study of patients with progressive, symptomatic, or recurrent DT who were randomized to treatment with the TKI sorafenib (inhibiting c-CRAF, BRAF, KIT, FLT-3, RET, RET/PTC, VEGFR-1, VEGFR-2, VEGFR-3, and PDGFR-β) versus placebo, an objective response rate of 33% using RECIST criteria was observed in the sorafenib group compared with a 20% objective response rate in the placebo group; 2-year progression-free survival rates were 81% versus 36%, respectively [26]. Grade 3/4 adverse events in the sorafenib arm included rash (14%), hypertension (8%), and fatigue (6%). The authors noted that the greater dose flexibility in this study may have reduced the high number of study withdrawals (20%). Patients in a phase 2 study of the TKI pazopanib (inhibiting VEGFR-1, -2, and -3; PDGFR-α-β; FGFR-1,3; c-KIT; ITK; LCK; c-FMS) versus methotrexate/vinblastine chemotherapy had a 41% response rate versus 30%, respectively, with 6-month progression-free survival rates of 85% in the pazopanib group versus 45% in the methotrexate/vinblastine group [27]. In the pazopanib arm, hypertension (21%) and diarrhea (15%) were the most common grade 3/4 adverse events, and 23% of patients had at least 1 drug-related serious adverse event.

The 2 AL101 cases presented here provide evidence for the activity of GSIs in patients with DT, with deep and sustained responses to treatment in patients with massive disease burden. In a phase 2 study of 17 patients with recurrent or unresectable DT, in which the majority of patients had a mutation in *CTNNB1* or *APC*, treatment with the GSI nirogacestat led to a PR rate of 29% [18]. An association between clinical response and mutation status was not made, perhaps due to the small sample size. Most patients experienced grade 1/2 diarrhea (76%) and skin toxicity (71%). Grade 3 hypophosphatemia (47%) was reported and was attributed to treatment with nirogacestat [18]. Among 4 pediatric patients with DT, 3 of whom had tumors harboring *APC* mutations [20], 3 patients (1 of whom did not have an *APC* mutation) had durable benefit with nirogacestat treatment. The fourth patient progressed on therapy after an initial PR. A phase 3 study is currently evaluating nirogacestat in adults with DT (NCT03785964).

The clear resistance to other systemic agents delivered prior to AL101 and the objective responses and response depth and duration in these 2 cases suggest that AL101, rather than spontaneous disease regression, led to the PRs (Figure 1). The ongoing maintenance of a PR in Case 1, which continues 4 years after discontinuation of AL101 therapy, is particularly intriguing and should be the subject of further investigation with this class of agents in the treatment of DT. Expanding on our limited sample of these 2 cases, in the context of a controlled clinical trial, will be valuable in determining the best treatment options for patients with DT.

Future research is required to optimize the treatment of patients with DT, while a clearer understanding of DT, the molecular determinants of progression/regression, and drug resistance in DT is critical to guide selection of the proper therapy of these patients. New targeted modalities, including inhibitors of nuclear β-catenin signaling and the gamma secretase of the Notch receptor, are on the horizon. A phase 2a expansion study of tegavivint, a nuclear β-catenin inhibitor that binds to transducin β-like protein 1, a downstream target in the WNT pathway, is underway in patients with progressive DT (NCT04851119) [28]. The pivotal phase 2/3 RINGSIDE study (NCT04871282) is examining an orally administered GSI, AL102, for the treatment of adults and adolescents with progressive DT; this study was initiated in March 2021 [29]. AL012 is structurally similar to AL101; however, this agent is administered orally. It has similar properties to AL101 with respect to the half-maximal inhibitory concentration necessary for inhibiting the cleavage of each of the Notch receptors, and like AL101, is a pan-Notch inhibitor. The initial open-label phase 2 portion of this study will enroll up to 36 patients with progressive DT and randomize patients to 1 of 3 study arms: 1.2 mg daily, 2 mg twice weekly, and 4 mg twice weekly. The subsequent phase 3 double-blind portion of the study will randomize up to 156 patients with progressive disease to receive AL102 or placebo (2:1). AL102 has been previously studied in a phase 1 trial in advanced solid tumors (NCT01986218), where it had a favorable safety profile; a single patient in the study with DT achieved durable stable disease, with a decrease in tumor size of 16.5% after approximately 9 months of treatment [21]. 

## 4. Conclusions

The two patients in this report presented with massive disease burden. After being treated with AL101, these patients had improvements in quality of life and long-lasting clinical responses, which were maintained more than 8.6 years in Case 1 and 2.6 years in Case 2. With continued monitoring, 1 patient was able to discontinue AL101 after 4.6 years of treatment, while maintaining a PR, and the other patient has maintained a PR at a reduced AL101 dose. Our case reports suggest that targeting the Notch pathway with the GSI AL102 could be an effective treatment strategy in patients with DT. A phase 2/3 clinical trial has been initiated to further assess AL102’s safety and efficacy among desmoid tumor patients.

## Figures and Tables

**Table 1:** Significant events and important clinical findings.

Feature	Case 1 (from NCT01292655)	Case 2 (from Compassionate Access Program)
Age at start of AL101, years	42	27
Sex	Female	Female
Race	White	White
Past medical history	Nonsignificant	Nonsignificant
*CTNNB1* mutation	T41A	None
*APC* mutation	None	Present
Desmoid tumor		
Age of onset	Disease diagnosis (September 2011): 1 year and 2 months before the start of AL101 treatment	Disease diagnosis (September 2015): 1 year and 4 months before the start of AL101 treatment
Primary site	Mesenteric desmoid tumor ^a^	Pelvis ^b^
Other sites	None	Abdominal wall
Symptoms	Abdominal pain, anorexia	Abdominal discomfort
Prior treatment		
Surgery	No	Proctocolectomy and ileoanal pouch
Systemic therapy	Imatinib (1L), tamoxifen (2L), doxorubicin (3L)	Tamoxifen (1L), dacarbazine/doxorubicin (2L), methotrexate/vinblastine (3L)
AL101		
Dose	8.4 mg QW (C1–C21); 6 mg Q2W (C22–C25); 4 mg Q2W (C26–C60) ^c^	4 mg QW (C1); 2.4 mg QW (C2–C3); 2.4 mg Q2W (C4–C26); 2.4 mg Q3W (C27–C55)
Duration	C1–C60 ^c^ (1662 days = 4.6 years)	C1–C55, ongoing (as of Mar 2021: 1526 days = 4.2 years)
Coadministered antitumor agents	No	No
Tumor size prior to initiation of AL101 ^a^, mm	178 (Day −12)	191 (Day 1)
Maximal tumor response ^d^ (tumor size, mm [%])	105 (−41.01%)	76 (–60.21%)
Response	PR at Day 386/ Week 55/C14, D19	PR at Day 573/ Week 82/C21, D13
Duration of response, years	8.6+	2.6+
Treatment-related adverse events		
Diarrhea	Grade 3	Grade 3
Lymphopenia	Grade 3	―
Elevated LFTs	―	Grade 2
Fatigue	Grade 2	Grade 1
Nausea	Grade 2	Grade 2
Weight decreased	Grade 2	Grade 2
Weight increased	Grade 2	―
Muscular weakness	Grade 2	―
Hypoalbuminemia	Grade 2	―
Orbital edema	Grade 2	―
Periorbital edema	Grade 2	―
Furuncle	Grade 2	―
Dermoid cyst	Grade 2	―
Folliculitis	Grade 2	―
Basal cell carcinoma (SAE)	Grade 2	―

Notes: ^a^ A single mesenteric desmoid tumor was followed to assess response in Case 1. ^b^ Two lesions, one pelvic and one abdominal, were followed to assess response for Case 2. ^c^ At the close of the trial, the patient was transferred to a compassionate use program during which the patient received two additional doses (at C60, D22 and at C61, D8), extending the total treatment duration to 1690 days. ^d^ Maximal tumor response in Case 2 is a summation of the long axis of 2 lesions. Abbreviations: 1L, first-line; 2L, second-line; 3L, third-line; C, cycle; D, day; LFT, liver function test; PR, partial response; Q2W, every 2 weeks; Q3W, every 3 weeks; QW, once weekly; SAE, serious adverse event.

## Data Availability

All the available data are contained within the article.
